# Antioxidant Efficacy of Esculetin against Tert-Butyl Hydroperoxide-Induced Oxidative Stress in HEK293 Cells

**DOI:** 10.3390/cimb44120407

**Published:** 2022-11-29

**Authors:** Woo Kwon Jung, Su-Bin Park, Hwa Young Yu, Yong Hwan Kim, Junghyun Kim

**Affiliations:** Department of Oral Pathology, School of Dentistry, Jeonbuk National University, Jeonju 54896, Republic of Korea

**Keywords:** esculetin, tert-butyl hydroperoxide, reactive oxygen species, kidney dysfunction

## Abstract

Esculetin is an antioxidant and anti-inflammatory compound derived from coumarin. Oxidative stress can cause overproduction of reactive oxygen species (ROS), which can lead to the development of chronic kidney failure. In this study, human embryonic kidney 293 (HEK293) cells were treated with tert-butyl hydroperoxide (t-BHP) to determine the antioxidant effects of esculetin. HEK293 cells were treated with t-BHP to validate changes in cell viability, ROS production, and apoptosis, and then treated with esculetin to evaluate the changes. Changes in mRNA and protein levels were analyzed using a proteome kit, PCR, and Western blotting. Esculetin improved HEK293 cell viability and reduced apoptosis caused by t-BHP-induced oxidative stress. At the mRNA and protein levels, esculetin decreased pro-apoptotic factor expression as well as increased anti-apoptotic factor expression. The antioxidant efficacy of esculetin was validated when it inhibited the apoptosis caused by t-BHP-induced oxidative stress in HEK293 cells.

## 1. Introduction

Kidneys play an important role in blood pressure and volume regulation by maintaining electrolyte balance in body fluids and removing waste products from the body, thereby maintaining homeostasis. However, renal function decline can occur as a result of diabetic nephropathy (with symptoms such as glomerular hypertrophy, mesangial expansion, and basement membrane thickening) [1,2], kidney dysfunction (due to obesity-induced hypertension) [3], and chronic kidney disease-induced renal failure [4].

Reactive oxygen species (ROS) generated excessively as a result of oxidative stress is linked to kidney dysfunction diseases, such as diabetic nephropathy [5], chronic kidney disease [6], and hypertension [7]. It also causes glomerular podocyte loss [8] and endoplasmic reticulum (ER) stress injury to human renal glomerular endothelial cells [9], both of which contribute to kidney dysfunction. In addition, one study found that oxidative stress increases the proliferation of mesangial cells [10] and is linked to glomerulonephritis [11].

Numerous antioxidant drugs that inhibit ROS generation have been studied to treat kidney dysfunction [12,13]. Natural products and their derivatives are gaining popularity as antioxidant drugs. Because of their chemical structure, drugs derived from natural products have greater chemical diversity, higher stereochemical content, and lower hydrophobicity, making them ideal drugs and sources of drugs [14,15,16,17].

In animal models of acute kidney injury and renal cells, antioxidants have shown to alleviate ROS-induced oxidative damage [18,19]. In addition, antioxidants have been shown in in vitro and in vivo experiments to prevent oxidative stress caused by high glucose levels in podocytes [20] as well as in clinical studies to treat diabetic nephropathy [21].

Coumarin is a naturally occurring benzopyrone that acts as an antioxidant and ROS scavenger. Esculetin, a coumarin-derived compound, is a natural plant product found in *Atremisia capillaris*, *Citrus limonia*, and *Euphorbia lathyris*, as well as Cortex Fraxini. It is a synthetic drug with antioxidant, antitumor, and anti-inflammatory properties. Because esculetin has higher antioxidant activity than other coumarins, studies on its use as an antioxidant to combat ROS are being conducted [22,23,24,25,26,27].

Using tert-butyl hydroperoxide (t-BHP) as an oxidative stress inducer, we investigated the antioxidant efficacy of esculetin against renal dysfunction in a human embryonic kidney 293 (HEK293) cell injury model.

## 2. Materials and Methods

### 2.1. Cell Culture and MTT Assay

After growing HEK293 to 70 percent confluence using DMEM (WELGENE, Daegu, Republic of Korea) supplemented with 10% FBS (WELGENE) in 96-well plates, esculetin and t-BHP were added at the indicated concentrations for the indicated times. To evaluate cell viability, MTT reagent was used (Sigma Aldrich, St. Louis, MO, USA).

### 2.2. Measurement of ROS

2′,7′-dichlorodihydrofluorescein diacetate (DCF-DA; Sigma Aldrich, St. Louis, MO, USA) was used to detect intracellularly active oxygen. Following 3 h of treatment with t-BHP and esculetin, HEK293 cells were treated with 3 μM DCF-DA for 30 min and washed with HBSS (WELGENE). The DCF-DA-positive cells were validated using a fluorescence microscope (BX51, Oylmpus, Tokyo, Japan). Fluorescence intensity was measured by imageJ software (NIH, Bethesda, MD, USA)

### 2.3. Apoptosis Assay

A one-step TUNEL staining kit (Promega, Madison, WI, USA) was used for the detection of apoptotic cells. Apoptotic cells were assessed under a fluorescence microscope. In this assessment, TUNEL-positive apoptotic cells were identified using green fluorescence emitted by the cells under fluorescence microscope (BX51, Oylmpus, Tokyo, Japan). ImageJ software (NIH, Bethesda, MD, USA) was used to count TUNEL-positive cells.

### 2.4. Proteome Assay Kit

The apoptosis-related signaling pathways were analyzed using Proteome Profiler™ (R&D Systems, Wiesbaden, Germany). Protein expression levels were determined using an image analyzer (ATTO, Tokyo, Japan).

### 2.5. Real-Time PCR

TRIzolTM reagent (Invitrogen, Carlsbad, CA, USA) was used to extract total RNA from the collected cells. The primers used were as follows: Bax, 5′-AAA CTG GTG CTC AAG GCC C-3′ and 5′-CTT CAG TGA CTC GGC CA GG-3′; Bcl2, 5′-GAT AAC GGA GGC TGG GAT GC-3′ and 5′-TCA CTT GTG GCC CAG ATA GG-3′; Caspase-3, 5′-TTG GAC TGT GGG ATT GAG ACG-3′ and 5′-CGC TGC ACA AAG TGA CTG GA-3′; PARP, 5′-GCT TCA GCC TCC TTG CTA CA-3′ and 5′-TTC GCC ACT TCA TCC ACT CC-3′; β-actin, 5′-CTC ACC CTG AAG TAC CCC ATC-3′ and 5′-GGA TAG CAC AGC CTG GAT AGC A-3′. PCRs were performed using a MiniOpticon™ Real-Time PCR System (Bio-Rad, Hercules, CA, USA) and 2xSYBR^®^ Green PCR Master Mix (Enzo, New York, NY, USA). The results were normalized to β-actin levels. Three independent experiments were conducted in triplicate.

### 2.6. Western Blot Analysis

Western blotting was used to evaluate the expression levels of apoptosis-related proteins in HEK293 cells. The antibodies were anti-Bax, anti-Bcl2, anti-PARP, and anti-caspase-3 (Abcam, Waltham, MA, USA). Finally, a Western blotting detection kit (SuperSignal™ West Dura Extended Duration Substrate, ThermoFisher Scientific, Waltham, MA, USA) was used to observe the protein bands after treatment with an HRP-conjugated secondary antibody (Advansta, San Jose, CA, USA). Protein expression levels were determined using an image analyzer (ATTO, Tokyo, Japan).

### 2.7. Statistical Analysis

The mean and standard deviation are calculated from the data. Prism 8.0 (GraphPad, San Diego, CA, USA) was used to perform an ANOVA followed by a Tukey’s post-hoc test.

## 3. Results

### 3.1. Esculetin Inhibits t-BHP-Induced HEK293 Cell Injury

**Figure 1:**
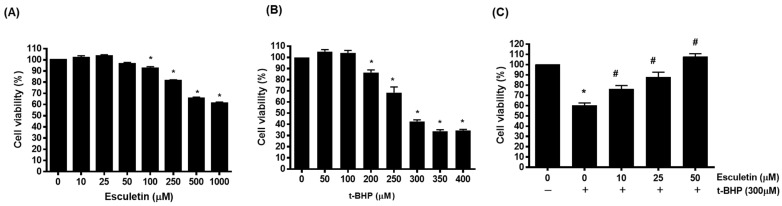
Effect of esculetin on t-BHP-induced HEK293 cell injury. The cell viability of HEK293 cells exposed to different concentrations of esculetin (**A**), t-BHP (**B**) and t-BHP with esculetin (**C**). In the bar graphs, the values represent means ± SD, *n* = 5. * *p* < 0.05 vs. untreated control group, # *p* < 0.05 vs. t-BHP-treated control group.

The effects of esculetin and t-BHP on HEK293 cells were confirmed using an MTT assay. Treatment with esculetin alone confirmed the concentration that did not affect cell viability, and there was no effect up to 50 μM (Figure 1A). t-BHP was found to decrease cell viability in a concentration-dependent manner, with cell viability being approximately 40% at 300 µM (Figure 1B). In addition, the combination of t-BHP and esculetin was found to increase cell viability in a concentration-dependent manner up to 50 μM (Figure 1C).

### 3.2. Esculetin Inhibits t-BHP-Induced ROS Generation in HEK293 Cells

**Figure 2:**
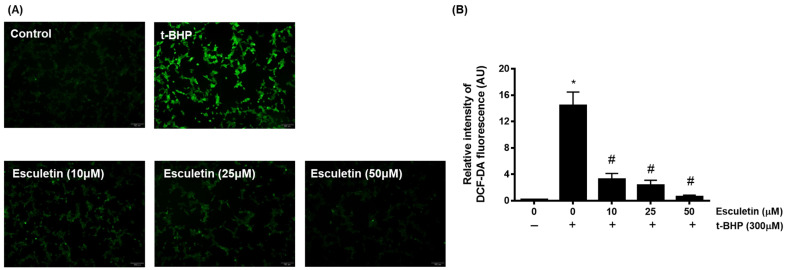
Effect of esculetin on t-BHP-induced ROS generation in HEK293 cells. (**A**) The changes in ROS levels in HEK253 cells exposed to t-BHP with esculetin were detected using DCFH-DA dye. (**B**) In the bar graphs, the values represent means ± SD, *n* = 5. * *p* < 0.05 vs. untreated control group, # *p* < 0.05 vs. t-BHP-treated group.

The effects of esculetin and t-BHP on HEK293 cells were confirmed using a DCF-DA assay. It was confirmed that t-BHP at 100 µM concentration promoted ROS generation in HEK293 cells, whereas esculetin inhibited it in a dose-dependent manner (Figure 2).

### 3.3. Esculetin Inhibits t-BHP-Induced Apoptosis of HEK293 Cells

**Figure 3:**
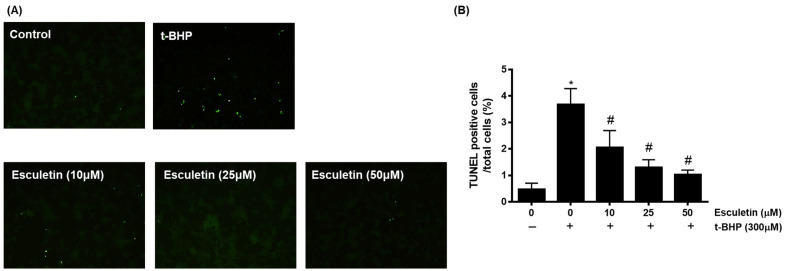
Effect of esculetin on t-BHP-induced apoptosis in HEK293 cells. (**A**) Apoptosis of HEK253 cells exposed to t-BHP with esculetin were detected using TUNEL staining. (**B**) In the bar graphs, the values represent means ± SD, *n* = 5. * *p* < 0.05 vs. untreated control group, # *p* < 0.05 vs. t-BHP-treated group.

TUNEL staining was used to confirm apoptosis by detecting cell death-associated DNA fragmentation by endonucleases [28]. It was confirmed that t-BHD promoted apoptosis, whereas esculetin inhibited it in a dose-dependent manner (Figure 3).

### 3.4. Esculetin Regulates Apoptosis-Related Signaling Pathways in HEK293 Cells

**Figure 4:**
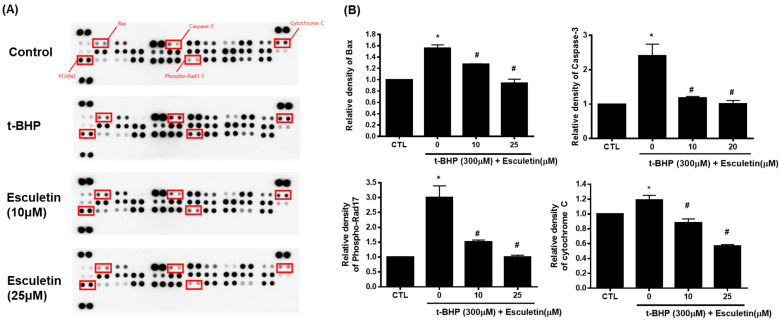
Effect of esculetin on apoptosis-related signaling pathways in HEK293 cells. (**A**) Apoptosis-related protein assay. (**B**) In the bar graphs, the values represent means ± SD, *n* = 5. * *p* < 0.05 vs. untreated control group, # *p* < 0.05 vs. t-BHP-treated group.

The Proteome Profiler Human Apoptosis Array Kit was used to validate the enriched signaling pathways. Cytochrome c, cleaved caspase-3, Bax, and phospho-Rad17 levels were increased by t-BHP and decreased by 10 µM esculetin (Figure 4).

### 3.5. Esculetin Regulates the Expression of Apoptosis-Related mRNA in HEK293 Cells

**Figure 5:**
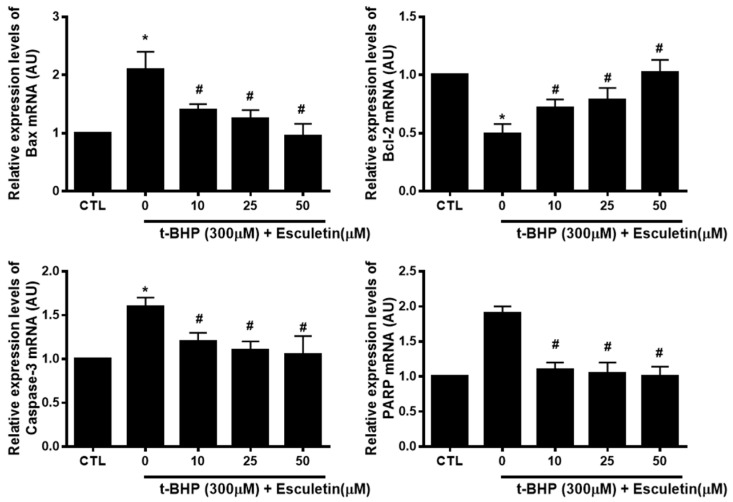
Effect of esculetin on the expression of apoptosis-related mRNA in HEK293 cells. The mRNA expression levels of Bax, bcl-2, caspase-3 and PARP. In the bar graphs, the values represent means ± SD, *n* = 5. * *p* < 0.05 vs. untreated control group, # *p* < 0.05 vs. t-BHP-treated group.

The mRNA expression of t-BHP and esculetin-affected apoptosis markers in HEK293 cells were investigated to validate the results of the protein array. t-BHP increased and decreased the mRNA levels of pro-apoptosis markers (Bax, PARP, and Caspase-3) and anti-apoptosis marker (Bcl-2), respectively. Esculetin was confirmed to decrease the mRNA expression of apoptosis markers in a dose-dependent manner (Figure 5).

### 3.6. Esculetin Regulates the Expression of Apoptosis-Related Proteins in HEK293 Cells

**Figure 6:**
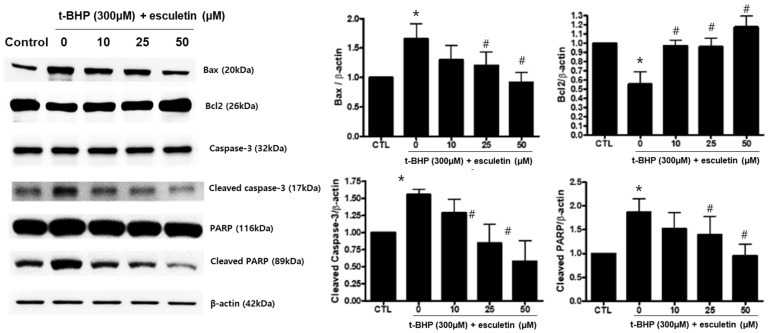
Effect of esculetin on the expression of apoptosis-related proteins in HEK293 cells. The protein expression levels of Bax, bcl-2, cleaved caspase-3 and cleaved PARP. In the bar graphs, the values represent means ± SD, *n* = 5. * *p* < 0.05 vs. untreated control group, # *p* < 0.05 vs. t-BHP-treated group.

The effects of t-BHP and esculetin on HEK293 cells were also confirmed at the protein level. t-BHP increased and decreased the protein expression of pro-apoptosis markers (Bax, PARP, and Caspase-3) and anti-apoptosis marker (Bcl-2), respectively. Esculetin was confirmed to decrease the protein expression of apoptosis markers in a dose-dependent manner (Figure 6).

## 4. Discussion

It was examined in the present study whether esculetin inhibits apoptosis induced by oxidative stress in HEK294 cells. There are several major pathogenic mechanisms involved in chronic kidney disease, such as damaged DNA repair systems, inflammation, increased apoptosis, and increased oxidative stress.

There are very low levels of ROS generated from the mitochondria of renal cells, but these levels can be significantly increased in response to certain factors, such as angiotensin II, TNF-α, LDL, and NADPH oxidase, as well as pathological conditions, such as diabetes [5,29].

Due to the presence of simian virus 40 large T antigen, HEK293 cells express a very high level of protein [30]. Human renal epithelial cells are frequently used to study oxidative stress, renal function, and renal diseases resulting from injury to renal epithelial cells [31].

Organic peroxide t-BHP induces oxidative stress via the peroxyl and alkoxyl radical pathways as well as the glutathione peroxidase pathway via cytochrome P450. It is generally used to investigate the effects of oxidative stress on cells and tissues. It has been used instead of H_2_O_2_ in oxidative stress studies [32,33]. In addition, t-BHP, a pro-oxidant compound, induces the production of free radicals via cytochrome P-450 and induces the generation of OH-radicals, such as lipid peroxides. ROS also inhibits cell proliferation by promoting oxidative stress-induced apoptosis [32,34,35,36].

Esculetin has been shown to be a potent antioxidant in various cells [37]. It reduces oxidative stress by inhibiting neutrophil-dependent superoxide anion production and lipid peroxidation, and by scavenging free radicals [38,39,40,41]. It has been demonstrated that esculetin reduces liver lesions induced by t-BHP, including hepatocellular edema, leukocyte infiltration, and necrosis in rats [41]. A hamster lung fibroblast cell (V79-4) treated with esculetin prevented lipid peroxidation, protein carbonylation, and DNA damage caused by H_2_O_2_ [42]. In oxidation-induced H9c2 cells, after esculetin treatment, Bcl-2 expression is up-regulated and Bax expression is down-regulated. Moreover, it inhibited the activity of caspase-3. As a result, esculetin improved viability in hypoxia/reoxygenation-stimulated H9c2 cells, suppressed oxidative stress, and inhibited cell death [43]. Furthermore, esculetin exerts anti-apoptosis activity in the mouse model of middle cerebral artery occlusion by up-regulating Bcl-2 expression and down-regulating Bax expression and downstream cleaving caspase-3 [44]. Based on these previous results, we confirmed that esculetin restores the proliferation of HEK293 cells that have been subjected to t-BHP-induced oxidative stress.

ROS-induced oxidative stress promotes endothelial changes that induce vascular remodeling (by promoting inflammation and enhancing cytokine production and expression of surface adhesion molecules) [45,46], induce apoptosis (via angiotensin II induction in renal proximal tubule epithelial cells (RPTECs) and mesangial cells), induce podocyte autophagy, and enhance ROS generation by promoting feedback loops [47,48,49]. Here, it was demonstrated that esculetin inhibits t-BHP-induced apoptosis of HEK293 cells. In addition, alterations in the apoptotic pathway caused by t-BHP-induced oxidative stress in HEK293 cells were confirmed at the mRNA and protein levels.

Anti-apoptotic proteins Bcl-2 and Bcl-XL inhibit cytochrome c release, whereas pro-apoptotic proteins, such as Bcl-2-associated X protein (Bax), Bcl-2 homologue antagonist/killer (Bak), and BH3 interacting-domain death agonist (Bid) promote cytochrome c release. The binding of cytochrome c to deoxyadenosine triphosphate (dATP) activates apoptotic protease activating factor (Apaf)-1 and procaspase-9. As a result, apoptosis is induced by caspase-3 activation [50,51,52].

Cytochrome c release is inhibited by anti-apoptotic proteins Bcl-2 and Bcl-XL, but promoted by pro-apoptotic proteins, such as Bax, Bak, and Bid. When cytochrome c binds deoxyadenosine triphosphate (dATP), apoptotic protease activating factor (Apaf)-1 and procaspase-9 are activated. Consequently, caspase-3 activation induces apoptosis [50,51,52].

Caspases are cysteine proteases that destroy cellular proteins and cause cell death. These caspase enzymes are classified as either initiators or effectors. Caspases-2,-8,-9, and-10 are apoptosis initiator caspases, while caspase-3,-6, and 7 are apoptosis effector caspases. In many tissues, caspase-3 promotes DNA fragmentation and cell death, and PARP-1 maintains genomic integrity and DNA repair. The caspase-3 enzyme cleaves PARP-1 and inactivates it during apoptosis. [53,54,55,56].

Studies have shown that the phenolic compound esculetin is an excellent antioxidant capable of reducing oxidative damage [57,58]. Another study found that esculetin reduced the oxidative stress marker increased by t-BHP in rat hepatocytes [41]. Furthermore, esculetin reduced apoptosis caused by H_2_O_2_-induced oxidative stress in C2C12 mouse myoblasts [59]. Similarly, in this study, esculetin inhibited pro-apoptotic proteins Bax, Caspase-3, and PARP-1, while activating anti-apoptotic protein Bcl2 in oxidative stress-induced apoptosis.

Therefore, esculetin was confirmed to inhibit apoptosis in HEK293 cells by reducing the t-BHP-induced oxidative stress.

As a result, esculetin mediated the cytoprotective effect of HEK293 cells under t-BHP-induced oxidative stress and reduced apoptosis, suggesting that it could be used as an antioxidant to alleviate renal dysfunction. However, while the efficacy of esculetin was demonstrated in vitro in this study, in vivo conditions could be altered by more complex mechanisms, necessitating additional in vivo studies.

## Data Availability

The data presented in this study are available on request from the corresponding author.
